# Analyzing and reducing common mode noise in high power inductive power transfer systems for electric vehicles using precise balance technique

**DOI:** 10.1038/s41598-025-31264-y

**Published:** 2025-12-08

**Authors:** Ying Mei, Wanying Weng, Jiande Wu, He Xu, Xiangning He, Yunfeng Wang

**Affiliations:** 1 College of Electrical Engineering, Zhejiang University, Hangzhou, 310027 China; 2 Midea Life Electric Appliances Co., Ltd, Foshan, 528311 China

**Keywords:** Balance technique, CM impedance, Common mode noise reduction, Distributed model, Inductive power transfer, Parasitic capacitance, Engineering, Electrical and electronic engineering

## Abstract

In inductive power transfer (IPT) charging systems for electric vehicles (EVs), shielding metals are commonly used to reduce electromagnetic field (EMF) radiation emitted by the coils. Nevertheless, these components also introduce additional common mode (CM) noise to the system and affect the electromagnetic compatibility (EMC) performance. To mitigate the impact of the CM noise, this paper investigates the asymmetric character of CM impedance of the IPT coils and proposes a distributed circuit model to reflect the stray capacitances of the IPT coils. A comprehensive analysis is conducted to determine the CM impedance and a complete CM noise model is subsequently derived for the IPT system. Based on the novel CM noise model, a balance technique is built on a symmetric compensation circuit topology, without the need for additional hardware. The balance technique is provided to ensure compliance with the CISPR 22 standard for CM noise. An 11 kW IPT prototype with the LCC (Inductor-Capacitor-Capacitor) compensation network has been implemented and experiments have been conducted. At low frequency (150 kHz to 5 MHz), the conductive CM noise is reduced by 5 dB; at high frequency (5 MHz to 30 MHz), is reduced by 13 dB, which validates the effectiveness of the proposed balance technique.

## Introduction

Inductive power transfer (IPT) stands out as the most popular approach for high-power wireless charging applications, such as unmanned aerial vehicles (UAVs), automated guided vehicles (AGVs) and especially EVs^1,2^. Meanwhile, a significant challenge arises from the broadband EM interference in high-power IPT-based EVs. Due to the complex structure of the EV charging system, many unintended parasitic parameters are generated in the circuit, including parasitic capacitances, inductances, and leakage flux. The conducted or radiated interference spreads through stray parameters in the EV charging system, leading to a decline in system performance and disruption to surrounding electrical equipment^3,4^. These challenges impede electromagnetic compatibility (EMC) and hinder popular research on the potential capabilities of integrating communication inside an IPT system^5^. Hence, the development of a precise EM noise transmission model and an effective EM suppression scheme is of utmost importance in high-power-based IPT systems.

**Fig. 1:**
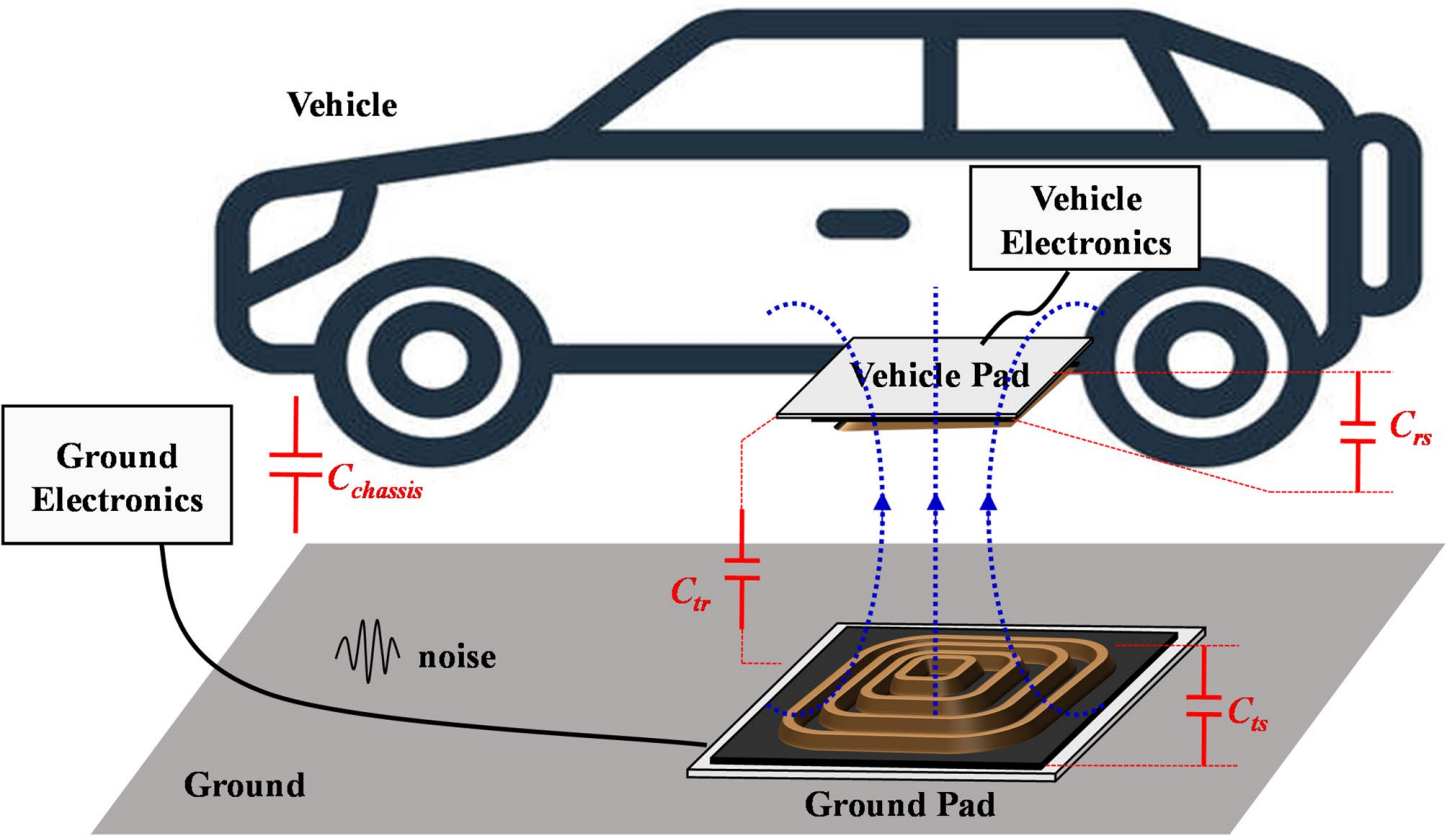
Parasitic capacitances in a typical IPT system for EV charging.

A typical IPT-based wireless EV charger is demonstrated in Fig. 1. The loosely coupled coils create low-frequency (85 kHz specified by SAE J2954 standard) EMF leakage to nearby environments. Besides, high dv/dt derived from fast-switching electronics can produce middle-frequency (150 kHz ~ 30 MHz) conductive emission and high-frequency (30 MHz ~ 1 GHz) radiation emission. The switching inverter in EVs generates noise sources that cause CM noise and contribute to the complex combined electromagnetic field spectrum^6,7^, so the mitigation of CM noise is always a concern issue.

In previous studies of CM noise, the effect of stray capacitances between power switches and heat sinks^8,9^, or stray capacitances between the transmitter and receiver coils^10,11^ had been extensively investigated. However, the parasitic capacitances arising from shielding metal were usually neglected^12,13^. This paper will illustrate the parasitic capacitances in IPT coils and their effect on the propagation of CM noise.

The high-power IPT system employs a metal shielding plate attached right below the ferrite layer of the coil pack. The shield minimizes the low-frequency EMF exposure for better EMC and safety. To avoid the induced floating voltage, the metal shielding layer shall be connected to the earth. Therefore, a significant parasitic capacitor between the transmitter IPT coil and the ground is introduced^14,15^, whilst the parasitic capacitor between transmitter and receiver coils can be disregarded^16^ due to the relatively large gap. Those parasitic capacitors will create a low-impedance path for CM noise, which is considerably unbalanced because of the complex characteristics of IPT coils. Thus, a distributed model is proposed to represent uneven features of stray capacitances. Consequently, an asymmetric CM impedance of two inverter nodes is derived based on the distributed coil model. Specific EM noise mitigation approaches must be implemented to meet rigorous EMC requirements of power and information transmission—CISPR 22. Conventional approaches adopt additional components, such as an EM interference (EMI) filter and an isolation transformer^17^. The passive EMI filter has the advantage of being highly reliable in solving EMI problems. However, its disadvantage is that it has a large volume and heavy weight^18^. Isolated transformers are commonly employed to provide galvanic isolation, thereby meeting safety requirements. However, transformers are the main propagation path for CM EMI noise caused by the high-frequency switching devices^7,19^. As a conclusion, these approaches could increase the material cost and lower the efficiency^3,8,9^.

Balanced approaches are commonly utilized in CM noise suppression. For instance^20^, proposes the use of balanced inverters to mitigate CM noise in PWM motor drives, while^21^ utilizes multilevel current source inverters to suppress CM voltage. However, implementing these approaches in an IPT system can significantly increase costs and introduce complexities in control. In an alternative approach^22^, suggests a strategy based on achieving balanced impedance in the Boost circuit. This strategy effectively suppresses CM noise without incurring additional costs.

This paper presents an asymmetrical distributed model of high-power IPT coils. Subsequently, a complete CM noise model is proposed based on the CM impedance analysis. Further, a novel balance technique utilizing a symmetrical compensation circuit topology is used to suppress CM noise without additional hardware. The design methodology of the compensation circuit is discussed in detail and the LCC network is utilized as an example. The methodology can also be extended to other circuit topologies. Finally, the experiments of an 11 kW IPT prototype validate that both low- and high-frequency CM noises are significantly reduced to satisfy the CISPR 22 standard.

## Common mode impedance of IPT coil

The asymmetric distribution of parasitic capacitances in an IPT coil is discussed and modeled in this section. For a more in-depth analysis of the distributed coil model and stray capacitance calculation, please refer to our previous research^14^. A comprehensive analysis to calculate the CM impedance of an IPT coil is introduced here. Comparison is made between the analytical results and measurements of an 11 kW IPT coil.

### Distributed coil model

**Fig. 2:**
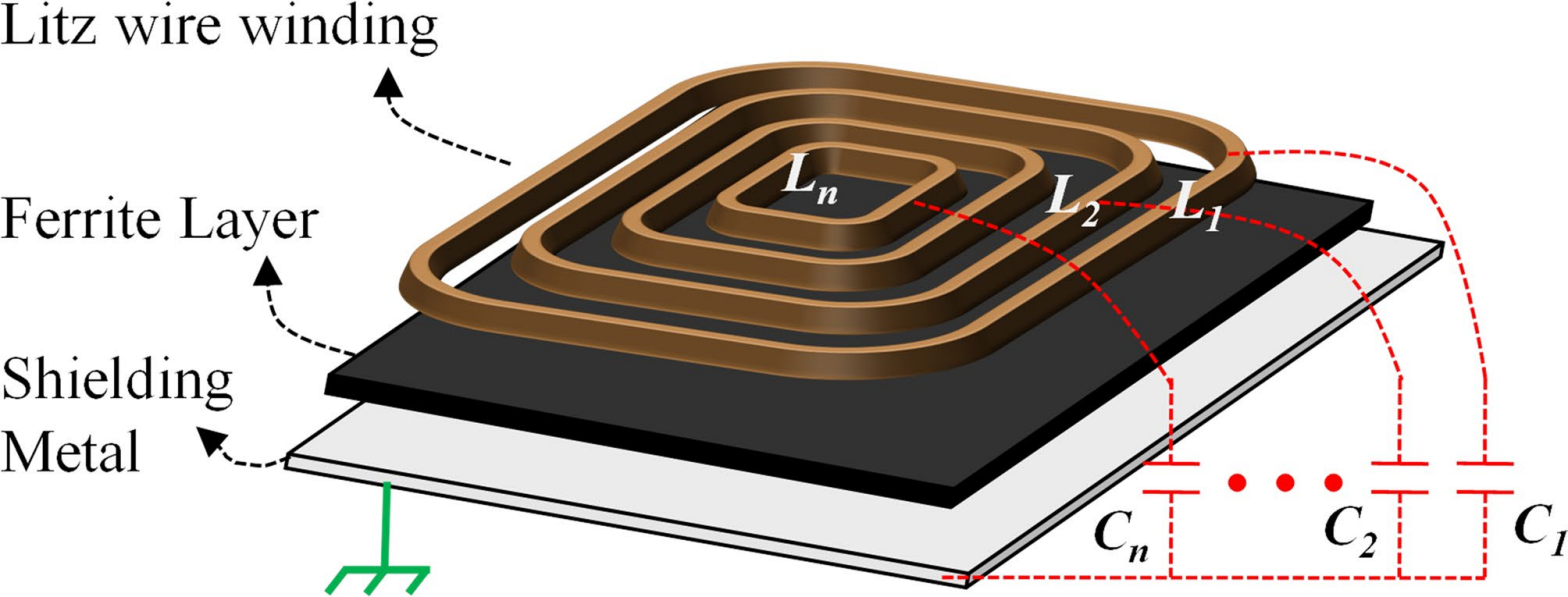
Comparison of parameters in IPT coil and traditional transformer.

Coupling coils are key components in an IPT system. Due to strict emission, heat dissipation, and safety concerns, a high-power coil is usually bulky and complicated. The coils behave similar to a loosely coupled transformer. In a wireless coil, each turn has different inductances and stray capacitances^10,23^, as shown in Fig. 2. Therefore, the CM impedances of two coil terminals is not equal. To reflect the asymmetric feature of an IPT coil, a distributed coil model is proposed.

**Fig. 3:**
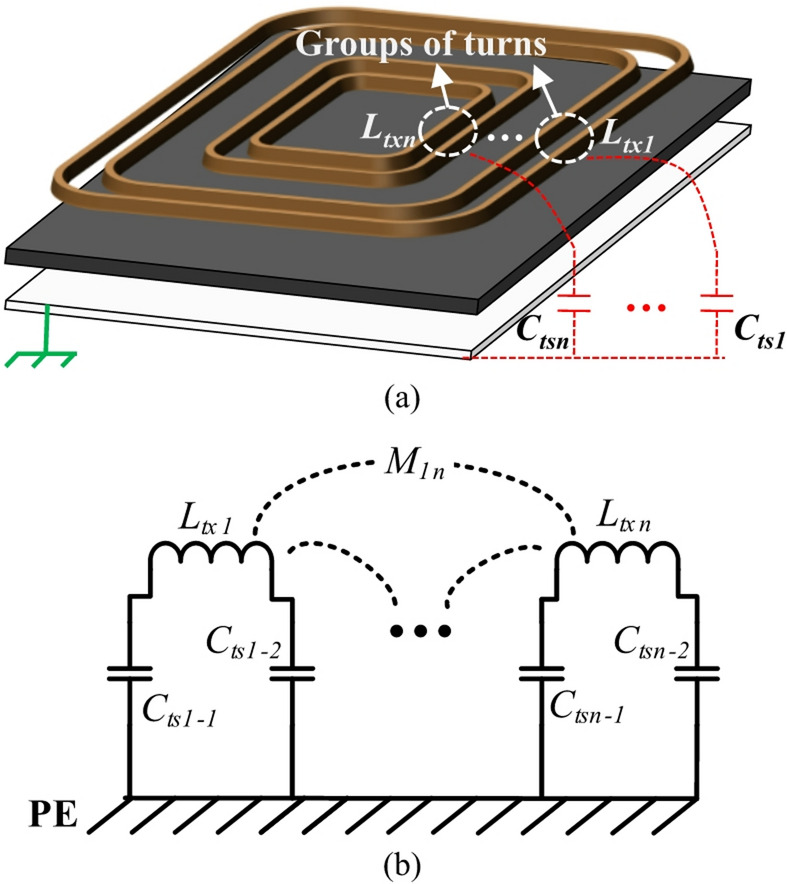
Distributed coil model of an IPT coil. (**a**) model concept (**b**) equivalent circuit.

However, separating the coil by the number of turns (as shown in Fig. 2) may present a problem of excessive turns, which complicates the analysis significantly. A multi-turn IPT coil is regarded as a collection of several individual conductors. In a practical design, not all the physical turns are modeled as a circuit unit. Instead, the planar coil is divided into several groups to reduce model complexity. As shown in Fig. 3a, each circuit unit has its self-inductance *L*_n_ and the stray capacitance to substrate *C*_sn_.

The equivalent circuit of the distributed model is illustrated in Fig. 3b. As can be observed in Fig. 3b, the simplest scenario of the distributed model is two conductors in series. That is, the transmitter coil with turn number *N* can be divided into two parts: the outer *N/2* turns of windings *L*_*tx1*_, and the inner *N/2* turns of windings *L*_*tx2*_. In the distributed coil model, the substrate stray capacitance is approximated as two identical capacitors connected to the input and output terminals of the distributed conductor, as illustrated in Fig. 3b. *C*_*ts1-1*_ represents half of stray capacitance between the outer *N/2* turns of windings and shielding metal. *C*_*ts2-2*_ represents half of stray capacitance between the inner *N/2* turns of windings and shielding metal. *C*_*ts1-2*_ and *C*_*ts2-1*_ respectively represent half of the stray capacitance between the shielding metal and the outer and inner *N*/2 winding turns. To simplify notation, *C*_*ts1*_ and *C*_*ts3*_ are defined as *C*_*ts1-1*_ and *C*_*ts2-2*_, respectively. Thus, *C*_*t2*_ = *C*_*ts1–2*_ + *C*_*ts2–1*_ = *C*_*ts1*_ + *C*_*ts3*_.

### Turn-to-shield Stray capacitance calculation

To estimate turn-to-shield stray capacitance in the distributed coil model, we simplified the shape of Litz wire, ferrite layer, and shielding layer as regular for calculation. More detailed comparison between the analytical capacitance calculation and FEM simulation can be found in our previous research^14^. Assume the fringe capacitance is negligible, the elementary capacitance between two opposite elementary surfaces of these conductors^23,24^ is given as below,

1 $$dC=\varepsilon \frac{dS}{x},$$

where, $\varepsilon = {\varepsilon }_{r}{\varepsilon }_{0}$ is the permittivity of gap medium and $x$ is the distance between two opposite elementary surfaces.

**Fig. 4:**
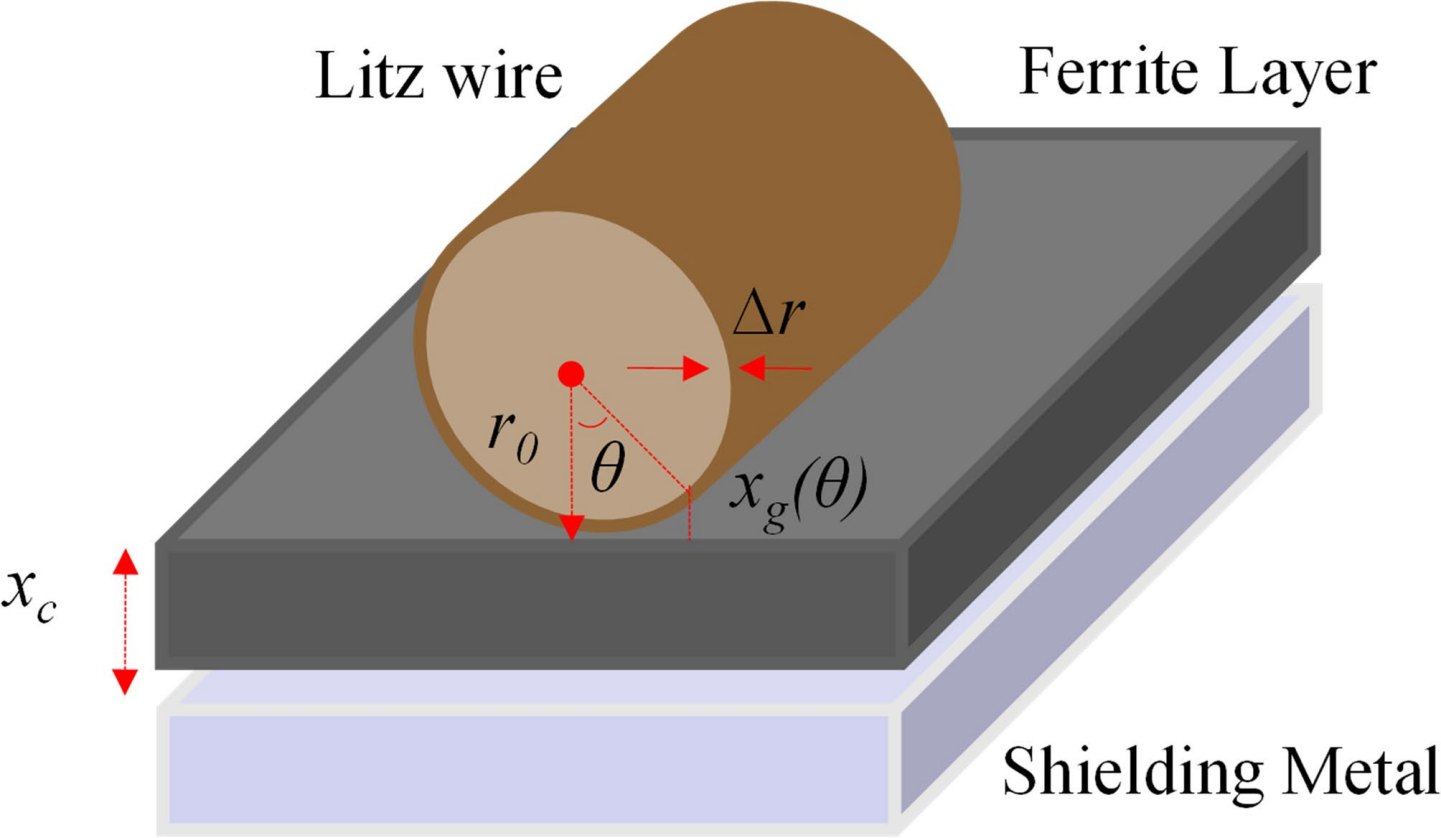
Cross-section view of an IPT coil pack.

Figure 4 shows the cross-sectional view of an IPT coil with round shape Litz-wire. It illustrates that the gap distance $x$ is not a constant. For easy calculation, the stray capacitance of a round shape wire is divided into two parts: air gap capacitance and ferrite core capacitance. The total capacitance equals the series combination of these two parts. The wire-diameter, insulator thickness, and ferrite layer thickness are defined as ${r}_{0}$, $\Delta r$, and ${x}_{c}$.

Distance between winding conductor and ferrite surface depends on the angular coordinate $\theta$, expressed as below,

2 $${x}_{g}\left(\theta \right)={r}_{0}\left(1-cos\theta \right)+\Delta r.$$

The elementary surface of a winding conductor per unit angle is given by,

3 $$dS={l}_{w}{r}_{0}d\theta ,$$

where ${l}_{w}$ is the length of Litz wire.

The air gap capacitance is obtained by integrating (1),

4 $${C}_{gap}=2{\int }_{0}^{\raisebox{1ex}{$\pi $}\!\left/ \!\raisebox{-1ex}{$2$}\right.}\frac{{\varepsilon }_{0}\bullet S}{{x}_{g}\left(\theta \right)}d\theta =2{\int }_{0}^{\raisebox{1ex}{$\pi $}\!\left/ \!\raisebox{-1ex}{$2$}\right.}\frac{{\varepsilon }_{0}\bullet {l}_{w}{r}_{0}}{{r}_{0}\left(1-cos\theta \right)+\Delta r}d\theta \approx 2{\varepsilon }_{0}{l}_{w}\left(\frac{{r}_{0}}{\Delta r}-1\right),$$

Ferrite core capacitance is also calculated as below,

5 $${C}_{core}=\frac{{\varepsilon }_{0}{\varepsilon }_{c}S}{{x}_{c}}=\frac{{\varepsilon }_{0}{\varepsilon }_{c}2{r}_{0}{l}_{w}}{{x}_{c}}.$$

Then, the total stray capacitance is calculated as below,

6 $${C}_{stray}=\frac{{C}_{gap}{C}_{core}}{{C}_{gap}+{C}_{core}}=\frac{2{\varepsilon }_{0}{\varepsilon }_{c}{r}_{0}{l}_{w}({r}_{0}-\Delta r)}{{\varepsilon }_{0}{r}_{0}\Delta r+{x}_{c}({r}_{0}-\Delta r)},$$

where ${\varepsilon }_{c}$ is the relative permittivity of ferrite.

In our previous work^14^, the theoretical stray capacitance, calculated using the formula above, was previously validated through simulation, demonstrating the model’s accuracy. Generally, the self-inductance and stray capacitance of each winding group are proportional to the length of winding. As a result, the self-inductances and stray capacitances of different loop windings are not distributed uniformly. This asymmetric characteristic of coil inlet and outlet can be easily expressed by the distributed model.

### Asymmetric CM impedance analysis

**Fig. 5.**
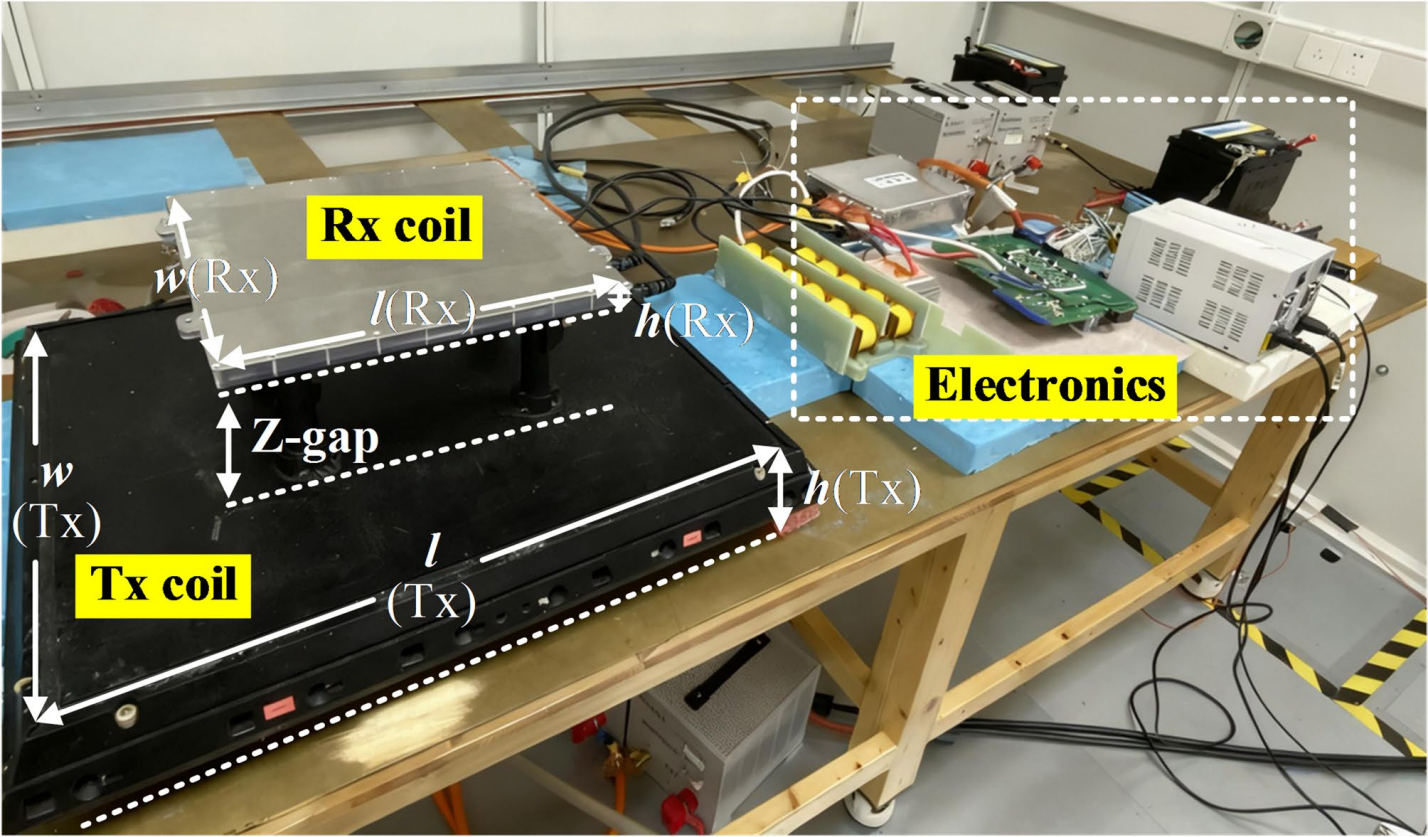
11 kW inductive coil prototype.

As shown in Fig. 5, a pair of 11 kW inductive coils are built for a case study, the targeted application is a wireless EV charger. An impedance analyzer (Keysight E4990A) is used for CM impedance measurement. The transmitter and receiver coils include three layers: the Litz wire layer, ferrite layer, and aluminum shielding layer. Table 1 lists the detailed parameters of the coil prototype.

**Table 1:** Design parameters of 11kw IPT coil prototype.

Parts	Layer name	Dimension (*l* × *w* × *h*) in mm	Material
Tx Coil	Litz wire layer	500 × 600 × 7	0.12 mm × 2000 strand
	Ferrite layer	520 × 620 × 8	DMR95 Chinadmegc
	Shielding layer	550 × 650 × 2	Aluminum
Z-gap	/	140 –210	Air
Rx Coil	Litz wire layer	310 × 310 × 5	0.12 mm × 1000 strand
	Ferrite layer	330 × 330 × 6	DMR95 Chinadmegc
	Shielding layer	350 × 350 × 2	Aluminum

**Fig. 6:**
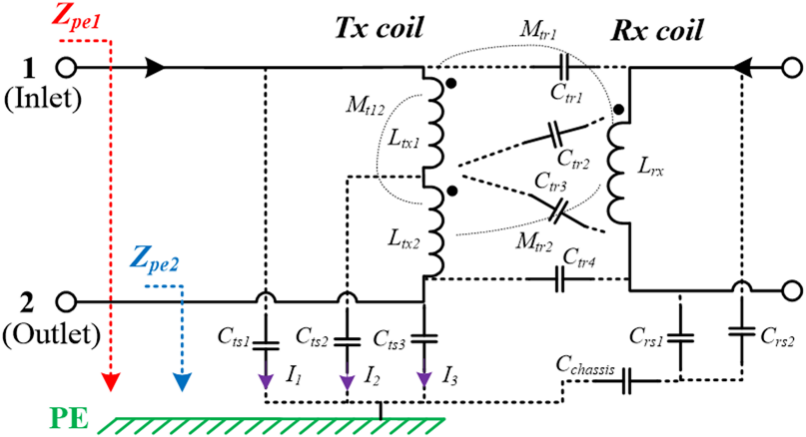
CM impedance with 2-sector distributed coil model.

The CM impedance model of the transmitter and receiver coils is shown in Fig. 6. The 2-sector distributed coil model is derived based on the analysis presented in Section "Distributed coil model" and illustrated in Fig. 3. On the transmitter side, there are distributed stray capacitances between transmitter coil and grounded metal-shielding layer, labeled as *C*_*ts1*_, *C*_*ts2*_, and *C*_*ts3*_. Due to relatively large distance between transmitter coil and receiver coil, the stray capacitances *C*_*ts1*_, *C*_*ts2*_ and *C*_*ts3*_ play a dominant role in CM noise propagation path.

**Fig. 7:**
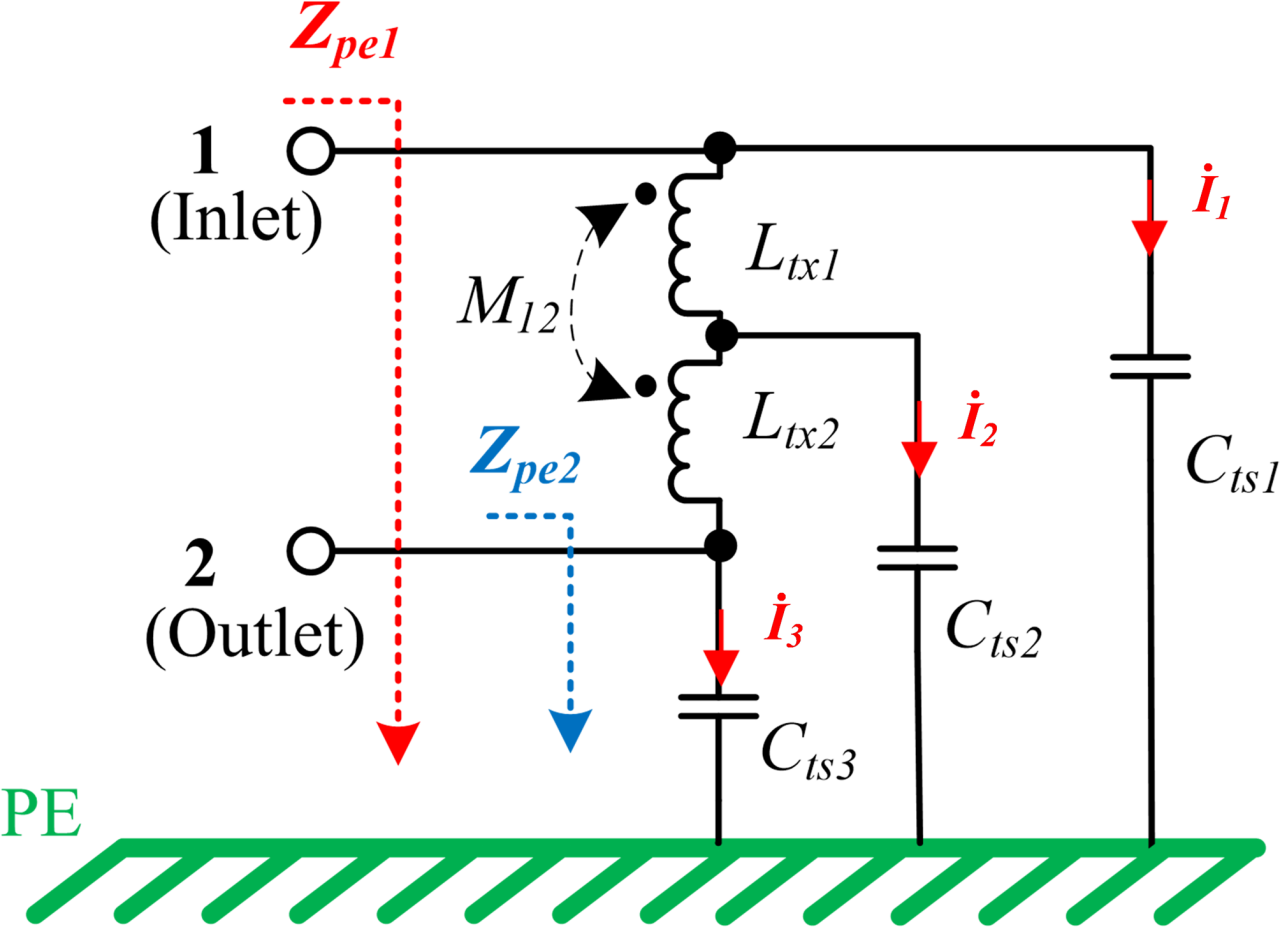
Simplified circuit without Rx components.

Two coil nodes are denoted as inlet and outlet respectively. As the uneven inlet and outlet stray capacitance features are depicted, the CM impedance of inlet (*Z*_*pe1*_) and outlet (*Z*_*pe2*_) is asymmetric. According to Table 1, the Z-gap between the Tx and Rx coils is much larger than the thickness of the Tx and Rx coils. As the distance increases, the value of the stray capacitance decreases rapidly. In our measurements, the stray capacitances between the Tx and Rx coils (*C*_*tr1-4*_) are typically minimum than 1 pF, which are relatively small when compared to the stray capacitances between the Litz wire layer and shielding layer (*C*_*ts1-3*_ in Table 2). So *C*_*tr1-4*_ can be neglected^14,16^ and the CM impedance model is simplified as Fig. 7.

**Table 2:** Components in two-section distributed model.

Symbol	Description	Typical estimated values
*C*_*inv1*_	Capacitance between power switch	200pF
*C*_*inv2*_	Capacitance between power switch	201pF
*C*_*ts1*_	Stray capacitance to Tx shielding-1	420pF
*C*_*ts2*_	Stray capacitance to Tx shielding-2	753pF
*C*_*ts3*_	Stray capacitance to Tx shielding-3	314pF
*L*_*tx1*_	Self-inductance of Tx coil outer part	66uH
*L*_*tx2*_	Self-inductance of Tx coil inner part	53uH
*L*_*rx*_	SELF-inductance of Rx coil	133uH
*M*_*t12*_	mutual inductance of *L*_*tx1*_ and *L*_*tx2*_	31uH
*M*_*tr1*_	Mutual inductance of *L*_*tx1*_ and *L*_*rx*_	13uH
*M*_*tr2*_	Mutual inductance of *L*_*tx2*_ and *L*_*rx*_	10uH

The simplified distributed model includes two conductors in series, as shown in Fig. 7. An N-turns transmitter coil is divided into two parts: the outer N/2-turn of windings *L*_*tx1*_, and the inner N/2-turn of windings *L*_*tx2*_. According to (4–6), three parasitic capacitances between the transmitter coil and metal-shielding layer (*C*_*ts1-3*_) are not the same. The typical estimated values of coil inductances and stray capacitances are listed in Table 2.

Based on the simplified distributed CM impedance model, the noise impedance introduced from coil inlet or outlet can be described separately in (7) and (8),

7 $$\left\{\begin{array}{c}{\dot{V}}_{CM1}=\frac{{\dot{I}}_{1}}{j\omega {C}_{ts1}}\\ {\dot{V}}_{CM1}=j\omega {L}_{tx1}\left({\dot{I}}_{2}+{\dot{I}}_{3}\right)+j\omega {M}_{t12}{\dot{I}}_{3}+\frac{{\dot{I}}_{2}}{j\omega {C}_{ts2}}\\ \frac{{\dot{I}}_{2}}{j\omega {C}_{ts2}}=j\omega {L}_{tx2}{\dot{I}}_{3}+j\omega {M}_{t12}\left({\dot{I}}_{2}+{\dot{I}}_{3}\right)+\frac{{\dot{I}}_{3}}{j\omega {C}_{ts3}}\\ {Z}_{pe1}=\frac{{\dot{V}}_{CM1}}{{\dot{I}}_{1}+{\dot{I}}_{2}+{\dot{I}}_{3}}\end{array},\right.$$

8 $$\left\{\begin{array}{c}{\dot{V}}_{CM2}=\frac{{\dot{I}}_{3}}{j\omega {C}_{ts3}}\\ {\dot{V}}_{CM2}=j\omega {L}_{tx2}\left({\dot{I}}_{1}+{\dot{I}}_{2}\right)+j\omega {M}_{t12}{\dot{I}}_{1}+\frac{{\dot{I}}_{2}}{j\omega {C}_{ts2}}\\ \frac{{\dot{I}}_{2}}{j\omega {C}_{ts2}}=j\omega {L}_{tx1}{\dot{I}}_{1}+j\omega {M}_{t12}\left({\dot{I}}_{1}+{\dot{I}}_{2}\right)+\frac{{\dot{I}}_{1}}{j\omega {C}_{ts1}}\\ {Z}_{pe2}=\frac{{\dot{V}}_{CM2}}{{\dot{I}}_{1}+{\dot{I}}_{2}+{\dot{I}}_{3}}\end{array}\right..$$

Furthermore, the analytical expressions of ${Z}_{pe1}$ and ${Z}_{pe2}$ can be derived from (7) and (8). Results are simplified in (9) and (10), where, ${L}_{tx}={L}_{tx1}+{L}_{tx2}+2{M}_{t12}$, $\Delta {L}_{t12}={L}_{tx1}{L}_{tx2}-{M}_{t12}^{2}$ and ${C}_{ts}={C}_{ts1}+{C}_{ts2}+{C}_{ts3}$, respectively.

9 $${Z}_{pe1}=-j\frac{1-{\omega }^{2}\left({C}_{ts2}{L}_{tx1}+{C}_{ts3}{L}_{tx}\right)+{\omega }^{4}{C}_{ts2}{C}_{ts3}\Delta {L}_{t12}}{\omega \left[{C}_{ts}-{\omega }^{2}\left({{C}_{ts1}C}_{ts2}{L}_{tx1}+{C}_{ts2}{C}_{ts3}{L}_{tx2}+{C}_{ts1}{C}_{ts2}{L}_{tx}\right)+{\omega }^{4}{{C}_{ts1}C}_{ts2}{C}_{ts3}\Delta {L}_{t12}\right]},$$

10 $${Z}_{pe2}=-j\frac{1-{\omega }^{2}\left({C}_{ts2}{L}_{tx2}+{C}_{ts1}{L}_{tx}\right)+{\omega }^{4}{C}_{ts1}{C}_{ts2}\Delta {L}_{t12}}{\omega \left[{C}_{ts}-{\omega }^{2}\left({{C}_{ts1}C}_{ts2}{L}_{tx1}+{C}_{ts2}{C}_{ts3}{L}_{tx2}+{C}_{ts1}{C}_{ts3}{L}_{tx}\right)+{\omega }^{4}{{C}_{ts1}C}_{ts2}{C}_{ts3}\Delta {L}_{t12}\right]}.$$

**Fig. 8:**
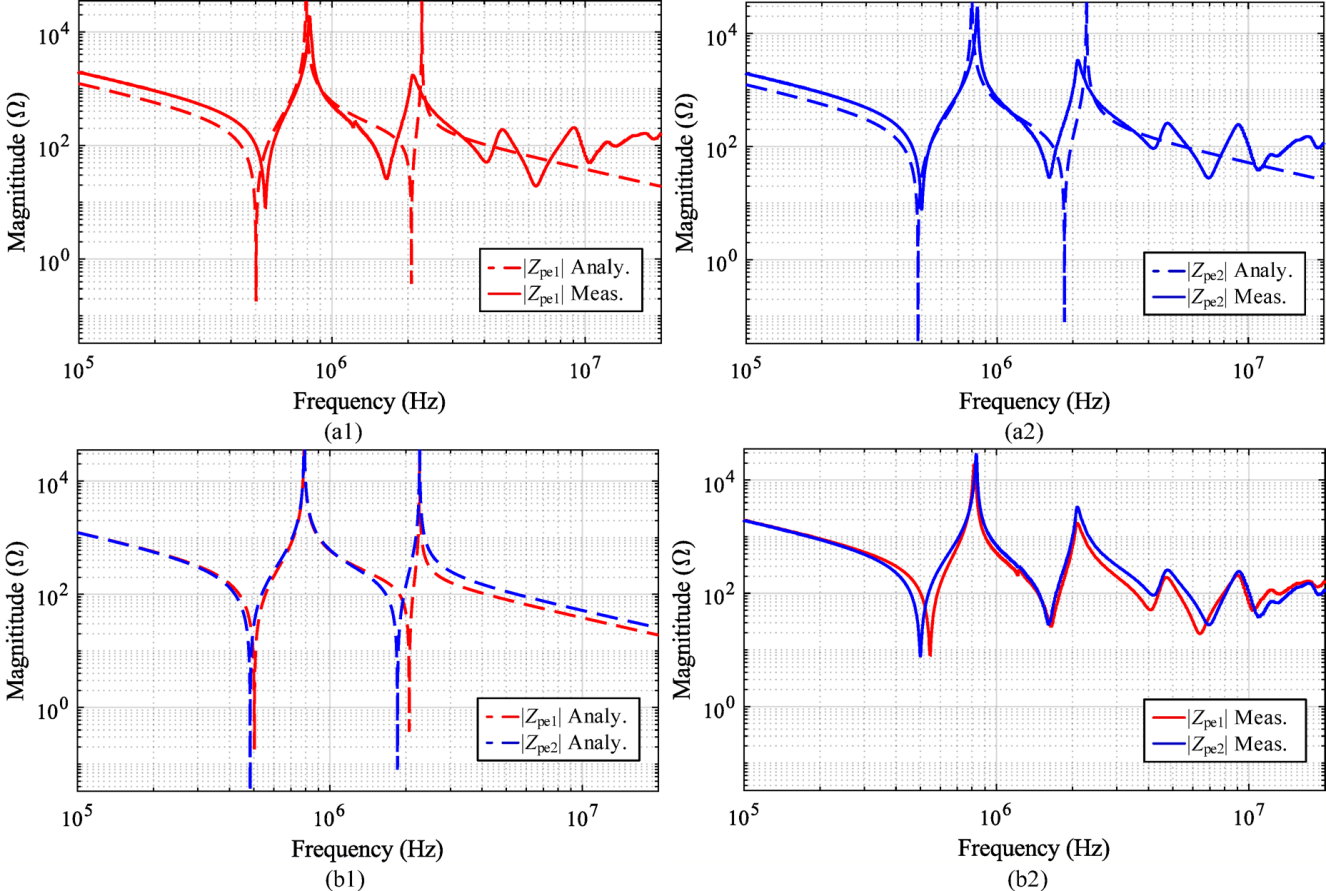
Analytical and measured results of CM impedance. (**a1**) Comparison of analytical *Z*_*pe1*_ and measured *Z*_*pe1*_ (**a2**) Comparison of analytical *Z*_*pe2*_ and measured *Z*_*pe2*_ (**b1**) Comparison of analytical *Z*_*pe1*_ and *Z*_*pe2*_ (**b2**) Comparison of measured *Z*_*pe1*_ and *Z*_*pe2*_*.*

The analytical CM impedances at the inlet and outlet nodes are derived in (9) and (10). To validate these expressions, both analytical and measured results for *Z*_*pe1*_ and *Z*_*pe2*_ are depicted in Fig. 8, with parameters listed in Table 2. In Fig. 8(a1), the analytical *Z*_*pe1*_ (dashed red lines) is compared with the measured *Z*_*pe1*_ (solid red lines). Although the curves’ match is not perfect, the discrepancy is primarily attributed to the complexity of high-frequency stray capacitances in practical scenarios. Nevertheless, the analytical results exhibit a similar trend to the measurements. Figure 8(a2) provides a similar comparison of *Z*_*pe2*_, where the analytical curve (dashed blue line) and the measured curve (solid blue line) are shown.

Furthermore, *Z*_*pe1*_ and *Z*_*pe2*_ are observed to be unbalanced, consistent with our earlier discussion. As illustrated in Fig. 8(b1) and (b2), both the analytical and measured *Z*_*pe1*_ differ from those of *Z*_*pe2*_. Since the analytical results and measured results are obtained under identical conditions, the observed differences confirm the imbalance between *Z*_*pe1*_ and *Z*_*pe2*_. In the following section, this unbalanced CM impedance model is used to analyze CM noise in the electrical circuit.

## Balance technique to suppress CM noise

In this section, the CM noise model of a high-power IPT system is studied. A balance technique is proposed to suppress the CM noise, which requires a symmetrical compensation network topology. A comprehensive design method of balanced CM impedance is presented based on an LCC-compensated IPT system. The feasibility of the proposed balanced impedance method is also verified by calculations and simulations.

### CM noise detection model of the IPT system

**Fig. 9:**
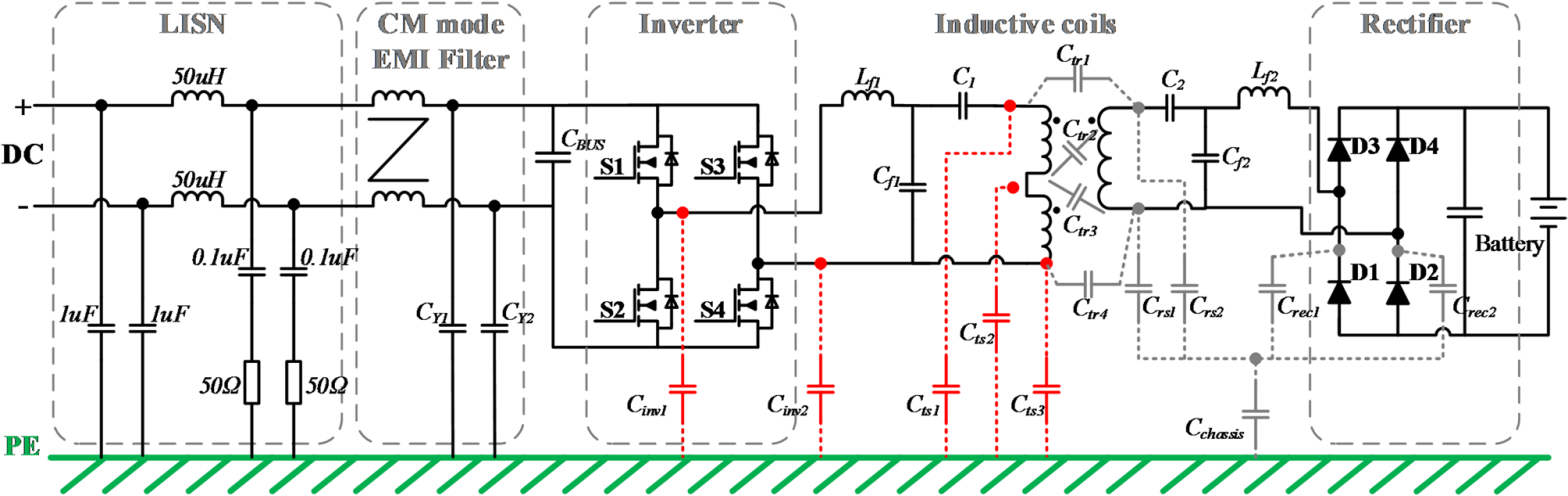
Circuit structure of an IPT system with double-side LCC compensation network.

A typical overall circuit structure of a high power IPT system with a double-side LCC compensation network is shown in Fig. 9. In the front end, there are EMI filter and PFC, which convert the utility ac power to dc power with power factor correction and EMI noise mitigation. There are a high-frequency inverter at the primary side and a diode rectifier as a secondary pick-up circuit. Double-side LCC circuits are adopted as the compensation network, which lead to a few advantages, including unit power factor, current source behavior, proportional output, and high efficiency^25^.

In the inductive coil pair, main parasitic capacitances (*C*_*ts1*_, *C*_*ts2*_, *C*_*ts3*_) are included according to the distributed coil model analyzed in section "Asymmetric CM impedance analysis". Moreover, due to the high-frequency operations in the inverter, parasitic capacitances between power switches and heat-sink (*C*_*inv1*_, *C*_*inv2*_) are taken into consideration. These parasitic capacitances are highlighted with red color. Similar parasitic capacitances also exist in the receiver coils; however, as elaborated in section "Asymmetric CM impedance analysis", the stray capacitances of the Rx have negligible influence on the CM noise path in the Tx, since the values of *C*_*tr1–4*_ are relatively small. Thus, the parasitic capacitances in the receiver coils are shown in gray, as they are not the focus of this study.

**Fig. 10:**
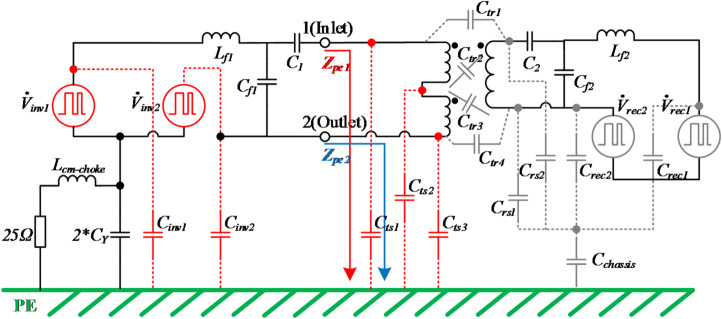
CM noise model of IPT system.

Based on the overall circuit structure, the CM noise model of the overall IPT system is presented in Fig. 10. Regarding high-frequency noises, the Line Impedance Stabilization Networks (LISNs) are modeled as two 50Ω resistors in parallel. The value of *L*cm-choke is generally determined by the additional insertion loss to meet the EMC standard. According to experience, it is between 10uH and 5mH. If it is too large, the cost and volume will be unbearable. If it is too small, there will not be enough insertion loss in the EMC frequency band of concern (150 kHz–30 MHz). This paper chooses 50uH as a reference. The CM noise is due to the voltage pulsating generated by high-frequency switching. The high value of dv/dt during switching generates CM noise currents flowing through parasitic capacitances from the IPT system to the ground. The switches are represented by four voltage sources as conductive CM noise sources: ${\dot{V}}_{inv1}$, ${\dot{V}}_{inv2}$, ${\dot{V}}_{rec1}$, and ${\dot{V}}_{rec2}$. As stated precedingly, the CM noise sources ${\dot{V}}_{rec1}$, and ${\dot{V}}_{rec2}$ are not the focus and shown in gray.

**Fig. 11:**
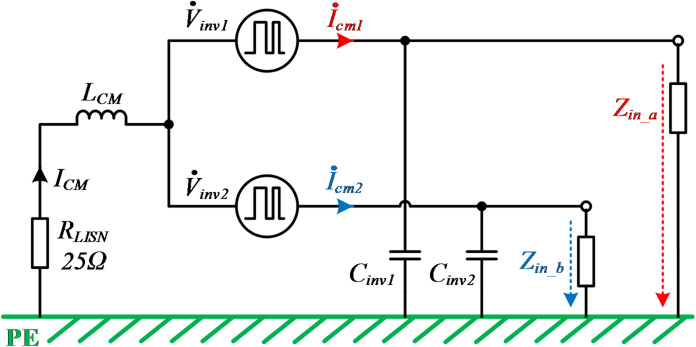
Simplified CM noise detection model at the primary side.

The CM noise circuit of the primary side in Fig. 10 mainly consists of CM noise sources and CM impedances. As analyzed in section "Asymmetric CM impedance analysis", *Z*_*pe1*_ and *Z*_*pe2*_ are not equivalent. In the entire IPT system, a compensation network is required and also influences the CM impedances. Figure 11 depicts the simplified CM noise model, where *Z*_*in_a*_ and *Z*_*in_b*_ represent the CM impedances incorporating the compensation network, specifically the LCC network adopted in this paper.

The CM current ${\dot{I}}_{CM1}$ and ${\dot{I}}_{CM2}$ excited by two noise sources (${\dot{V}}_{inv1}$, ${\dot{V}}_{inv2}$) are expressed in Eqs. (11) and (12),

11 $${\dot{I}}_{CM1}={\dot{V}}_{inv1}/[{(R}_{LISN}+j\omega {L}_{CM})||\left({Z}_{i{n}_{b}}\left|\left|\frac{1}{j\omega {C}_{inv2}}\right)+\left(\frac{1}{j\omega {C}_{inv1}}\right)\right||{Z}_{i{n}_{a}}\right],$$

12 $${\dot{I}}_{CM2}={\dot{V}}_{inv2}/[{(R}_{LISN}+j\omega {L}_{CM})||\left({Z}_{i{n}_{a}}\left|\left|\frac{1}{j\omega {C}_{inv1}}\right)+\left(\frac{1}{j\omega {C}_{inv2}}\right)\right||{Z}_{i{n}_{b}}\right].$$

The total CM noise ${\dot{I}}_{CM}$ is the sum of ${\dot{I}}_{CM1}$ and ${\dot{I}}_{CM2}$.

13 $${\dot{I}}_{CM}={\dot{I}}_{CM1}+{\dot{I}}_{CM2}.$$

Considering the symmetry of the full-bridge inverter, ${C}_{inv1}$ and ${C}_{inv2}$ can be considered to have the same value. Therefore, ${Z}_{in\_a}||\frac{1}{j\omega {C}_{inv1}}$ is denoted as ${Z}_{inac}$, ${Z}_{in\_b}||\frac{1}{j\omega {C}_{inv2}}$ is denoted as ${Z}_{inbc}$ and ${R}_{LISN}+j\omega {L}_{CM}$ is denoted as ${Z}_{LN}$, respectively.

Combined with (11) and (12), (13) can be further simplified as

14 $$\begin{aligned} \dot{I}_{{CM}} = \dot{I}_{{CM1}} + \dot{I}_{{CM2}} = & \;\frac{{\dot{V}_{{inv1}} }}{{Z_{{LN}} ||Z_{{inbc}} + Z_{{inac}} }} + \frac{{\dot{V}_{{inv2}} }}{{Z_{{LN}} ||Z_{{inac}} + Z_{{inbc}} }} \\ = & \;\frac{{Z_{{LN}} }}{{Z_{{inac}} + Z_{{inbc}} }} \cdot \frac{{\dot{V}_{{inv1}} + \dot{V}_{{inv2}} }}{{Z_{{LN}} + Z_{{inac}} ||Z_{{inbc}} }} + \frac{1}{{Z_{{inac}} + Z_{{inbc}} }} \cdot \frac{{Z_{{inbc}} \dot{V}_{{inv1}} + Z_{{inac}} \dot{V}_{{inv2}} }}{{Z_{{LN}} + Z_{{inac}} ||Z_{{inbc}} }}. \\ \end{aligned}$$

Since ${\dot{V}}_{inv2}$ is 180° phase lagging of ${\dot{V}}_{inv1}$, the CM noise can be canceled when the loop impedances are balanced (e.g. ${Z}_{inac}$ = ${Z}_{inbc}$). Thus, according to (14), the noise source of the system can be redefined as $\frac{1}{2}({\dot{V}}_{inv1}+{\dot{V}}_{inv2})$. Subsequently, the expression of the CM impedance ${Z}_{CM}$ of the balanced system can be obtained as

15 $${Z}_{CM}=\frac{\frac{1}{2}({\dot{V}}_{inv1}+{\dot{V}}_{inv2})}{{\dot{I}}_{CM}}={(Z}_{LN}+{Z}_{inac}|\left|{Z}_{inbc}\right)\left(\frac{{Z}_{inac}+{Z}_{inbc}}{{2Z}_{LN}}+1\right).$$

### Balanced impedance design method

The key CM noise propagation path depicted in Fig. 10 has been simplified to the circuit shown in Fig. 11. As can be found in Fig. 11, if the CM impedance is not balanced, it may cause more CM noise current to flow through the two bridge legs. Therefore, an impedance design method should be applied to balance the ground impedance ${Z}_{in\_a}$ and ${Z}_{in\_b}$ of the upper and lower bridge legs of the inverter. Even though the noise path is clear in Fig. 11, it is difficult to directly quantify ${Z}_{in\_a}$ and ${Z}_{in\_b}$.

**Fig. 12:**
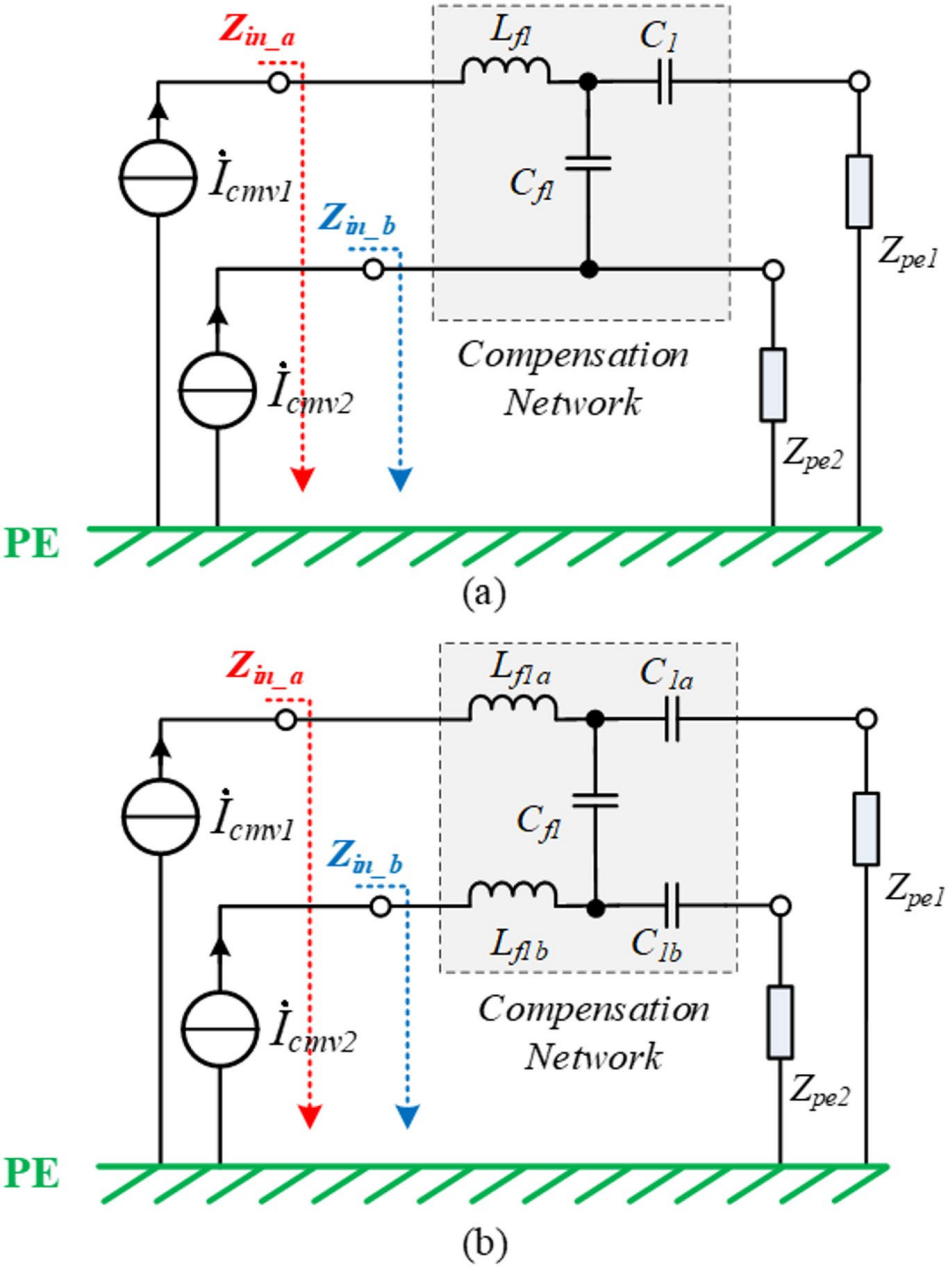
Compensation network design for balanced CM impedance. (**a**) with unbalanced CM impedance; (**b**) with balanced CM impedance.

Considering that ${Z}_{in\_a}$ and ${Z}_{in\_b}$ represent complex combinations of the LCC compensation network, ${Z}_{pe1}$, and ${Z}_{pe2}$. In previous sections, the uneven character of the Tx coil’s inlet and outlet impedances ${Z}_{pe1}$ and ${Z}_{pe2}$ is analyzed. Subsequently, the compensation network should be systemically designed according to Tx coil’s inlet and outlet features. In Fig. 11, similar source circuits can be further simplified. The CM currents ${\dot{I}}_{cmv1}$ and ${\dot{I}}_{cmv2}$ are conducted through the stray capacitors ${C}_{inv1}$ and ${C}_{inv2}$, which are considered similar. Therefore, by using the CM current sources instead of the stray capacitor branches, modeling of CM current path in the passive network, can be further simplified as shown in Fig. 12.

Figure 12a displays the equivalent circuit of a conventional compensation network. The resonant inductor ${L}_{f1}$ and resonant capacitor ${C}_{1}$ are allocated on one branch of two inverter legs. Then, the unbalanced component values of ${Z}_{inacu}$ and ${Z}_{inbcu}$ are derived by the following equations,

16 $${Z}_{inacu}={Z}_{in\_a}\left|\left|\frac{1}{j\omega {C}_{inv1}}=\left[j\omega {L}_{f1}+(\frac{1}{j\omega {c}_{1}}+{Z}_{pe1})||(\frac{1}{j\omega {c}_{f1}}+{Z}_{pe2})\right]\right|\right|\frac{1}{j\omega {C}_{inv1}},$$

17 $${Z}_{inbcu}={Z}_{in\_b}||\frac{1}{j\omega {C}_{inv2}}=(\frac{1}{j\omega {c}_{f1}}+\frac{1}{j\omega {c}_{1}}+{Z}_{pe1})||{Z}_{pe2}||\frac{1}{j\omega {C}_{inv2}}.$$

**Fig. 13:**
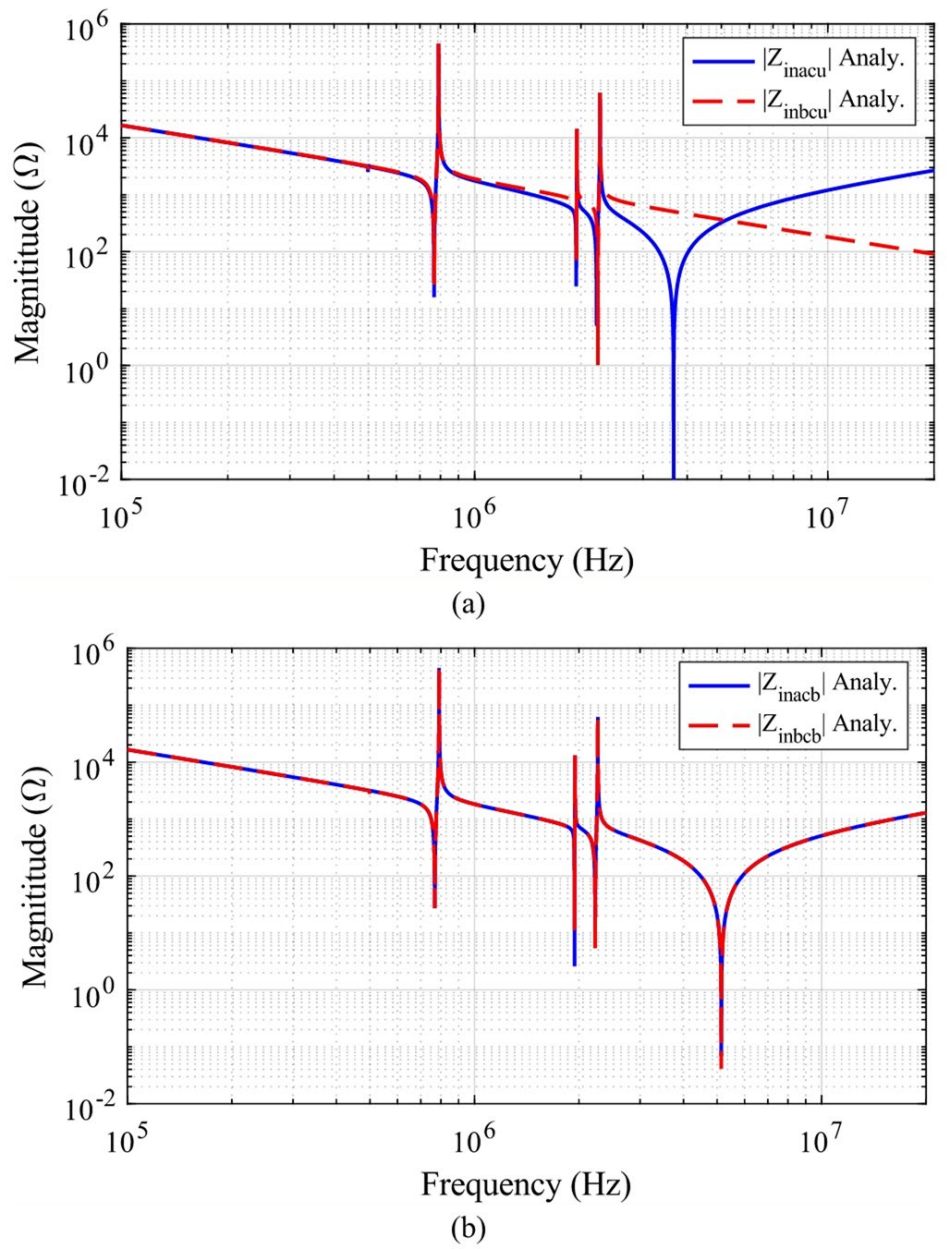
The equivalent impedance ${Z}_{inac}$ of upper bridge leg and the equivalent impedance ${Z}_{inbc}$ of lower bridge leg. (**a**) with unbalanced CM impedance; (**b**) with balanced CM impedance.

**Fig. 14:**
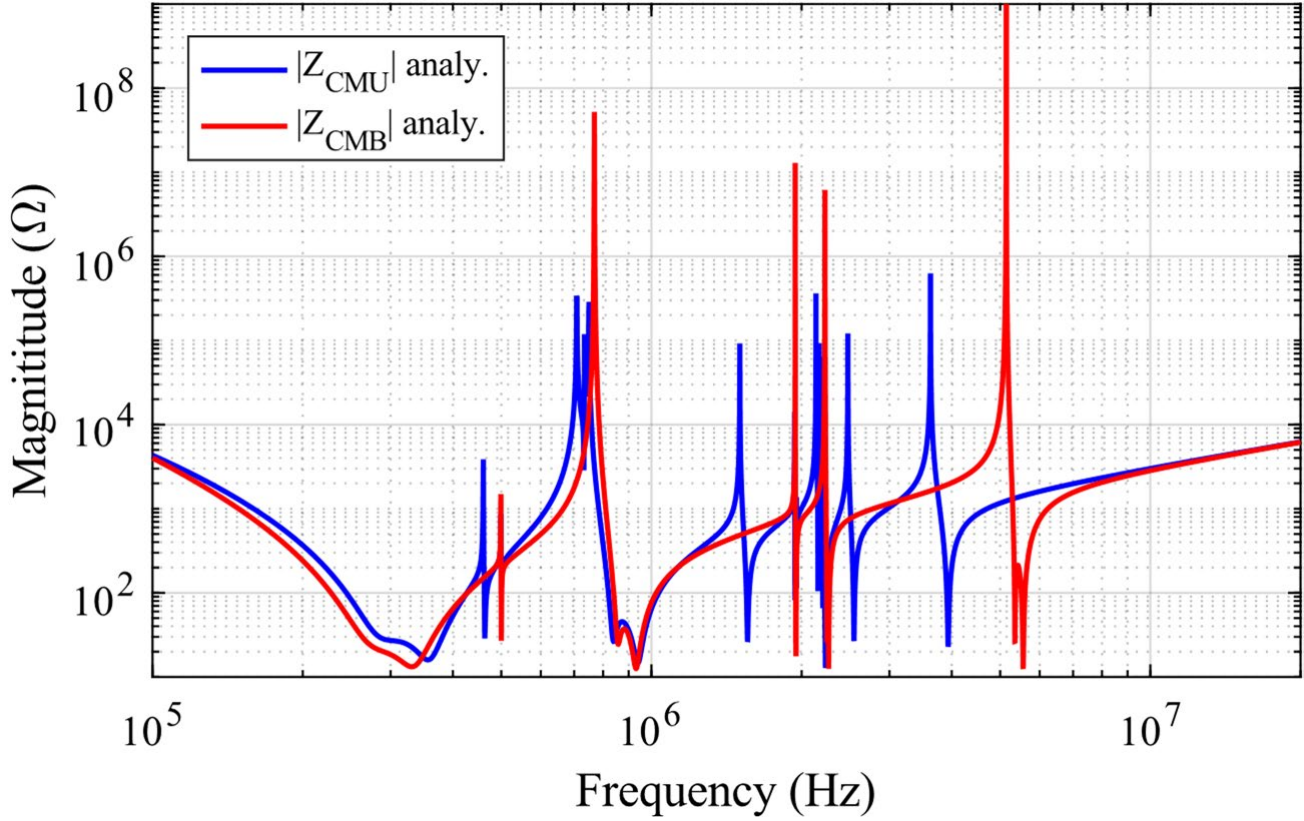
The comparison between the common-mode impedance ${Z}_{CMB}$ of the system by balanced impedance method and the ideal common-mode impedance ${Z}_{CMU}$.

Figure 13a shows the unbalanced component values of ${Z}_{inacu}$ and ${Z}_{inbcu}$, as described in (16) and (17). It is evident that ${Z}_{inacu}$ is not equal to ${Z}_{inbcu}$. Consequently, the direct calculation of the CM impedance ${Z}_{CMU}$ of the system using (15) is not possible before employing the balanced impedance method. However, in an extreme scenario where ${\dot{V}}_{inv1}$ and ${\dot{V}}_{inv2}$ have the same phase, the CM noise can be effectively canceled. In this case, the ideal CM impedance ${Z}_{CMU}$ of the system can be calculated using (15), and the corresponding results are depicted in Fig. 14 as a reference.

To achieve the balanced CM impedance, the compensation network needs to be rebuilt. The structure of the redesigned network is illustrated in Fig. 12b. The resonant inductor ${L}_{f1}$ is split into two inductors (${L}_{f1a}$, ${L}_{f1b}$), and the resonant capacitor ${C}_{1}$ is also split into two capacitors (${C}_{1a}$, ${C}_{1b}$).

18 $$\left\{\begin{array}{c}{Z}_{inacb}=\left[j\omega {L}_{f1a}+\left(\frac{1}{j\omega {C}_{1a}}+{Z}_{pe1}\right)||(\frac{1}{j\omega {C}_{1b}}+\frac{1}{j\omega {C}_{f1}}+{Z}_{pe2})\right]||\frac{1}{j\omega {C}_{inv1}}\\ {Z}_{inbcb}=\left[j\omega {L}_{f1b}+\left(\frac{1}{j\omega {C}_{1b}}+{Z}_{pe2}\right)||(\frac{1}{j\omega {C}_{1a}}+\frac{1}{j\omega {C}_{f1}}+{Z}_{pe1})\right]||\frac{1}{j\omega {C}_{inv2}}\\ {L}_{f1}={L}_{f1a}+{L}_{f1b}\\ \frac{1}{{C}_{1}}=\frac{1}{{C}_{1a}}+\frac{1}{{C}_{1b}}\end{array}\right..$$

The component value is derived in (18) to achieve the balanced CM impedance.

The resonant capacitor ${C}_{f1}$ mainly acts as a differential component, and its impact on CM impedance can be ignored. Many alternatives are available for (18). However, observing both sides of Eq. (18), for rapid prototyping, if each branch of ${Z}_{inacb}$ and ${Z}_{inbcb}$ are balanced, then ${Z}_{inacb}={Z}_{inbcb}$, the following conditions must be met,

19 $$\left\{\begin{array}{c}j\omega {L}_{f1a}=j\omega {L}_{f1b}\\ \frac{1}{j\omega {C}_{1b}}+\frac{1}{j\omega {C}_{f1}}+{Z}_{pe2}=\frac{1}{j\omega {C}_{1a}}+\frac{1}{j\omega {C}_{f1}}+{Z}_{pe1}\\ {L}_{f1}={L}_{f1a}+{L}_{f1b}\\ \frac{1}{{C}_{1}}=\frac{1}{{C}_{1a}}+\frac{1}{{C}_{1b}}\end{array}\right..$$

According to (19), ${L}_{f1a}$ is equal to ${L}_{f1b}$. Then, the values of resonant components are redesigned as below,

20 $$\frac{{L}_{f1}}{2}={L}_{f1a}={L}_{f1b},$$

21 $${C}_{1a}=\frac{2}{{1/C}_{1}+j\omega ({Z}_{pe2}-{Z}_{pe1})},$$

22 $${C}_{1b}=\frac{2}{{1/C}_{1}-j\omega ({Z}_{pe2}-{Z}_{pe1})}.$$

The component values of the original LCC compensation network and the adjusted network to achieve the balanced CM impedance are shown in Table 3. Figure 13b displays the balanced component values of ${Z}_{inacb}$ and ${Z}_{inbcb}$, as given in (18). It is evident that ${Z}_{inacb}$ is almost equal to ${Z}_{inbcb}$ when employing the balanced impedance method.

**Table 3:** Design parameters for 11 kW IPT transmitter coil.

Category	Symbol	Description	Value
Unbalanced impedance	*L*_*f1*_	Resonant inductance	22uH
	*C*_*f1*_	Parallel resonant capacitor	175nF
	*C*_*1*_	Series resonant capacitor	14.5nF
Balanced impedance	*L*_*f1a*_	Resonant inductance-a	11uH
	*L*_*f1b*_	Resonant inductance-b	11uH
	*C*_*f1*_	parallel resonant capacitor	175nF
	*C*_*1a*_	Series resonant capacitor-a	22nF
	*C*_*1b*_	Series resonant capacitor-b	43nF

At this time, the CM impedance ${Z}_{CMB}$ of the system using balanced impedance method can be calculated by (15), and the calculated result is shown in Fig. 14. Compared with the ideal CM impedance ${Z}_{CMU}$, the balanced impedance method can make the CM impedance ${Z}_{CMB}$ approach to ideal impedance ${Z}_{CMU}$, thus realizing the CM noise suppression without additional devices.

### Simulation study

**Fig. 15:**
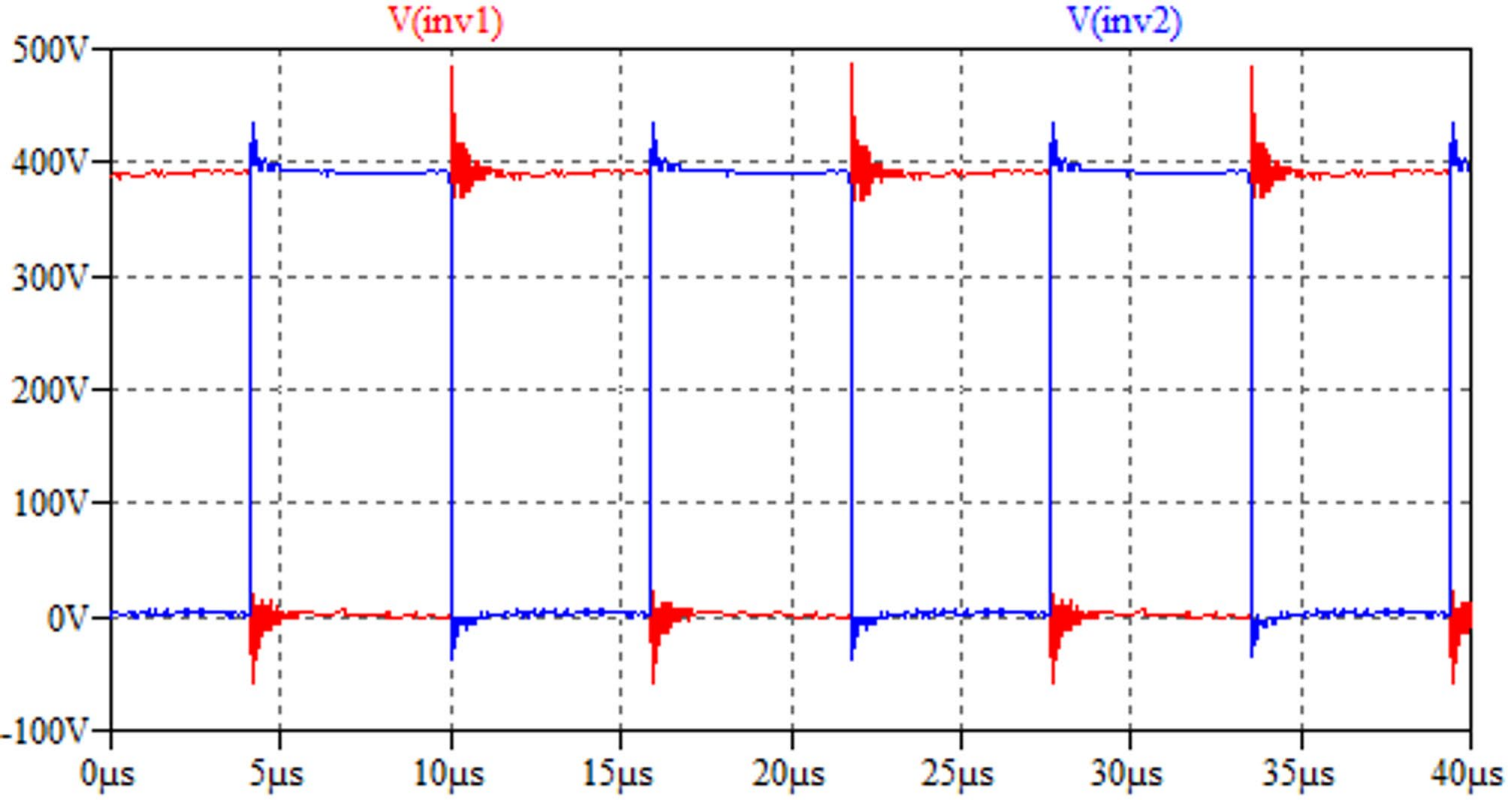
High-frequency switching noise sources used in LTSpice simulation.

The CM noise model in Fig. 11 is simulated by LTSpice. During the experimental test, the real waveforms of inverter nodes are captured and rebuilt as noise sources in the simulation model, as shown in Fig. 15.

**Fig. 16:**
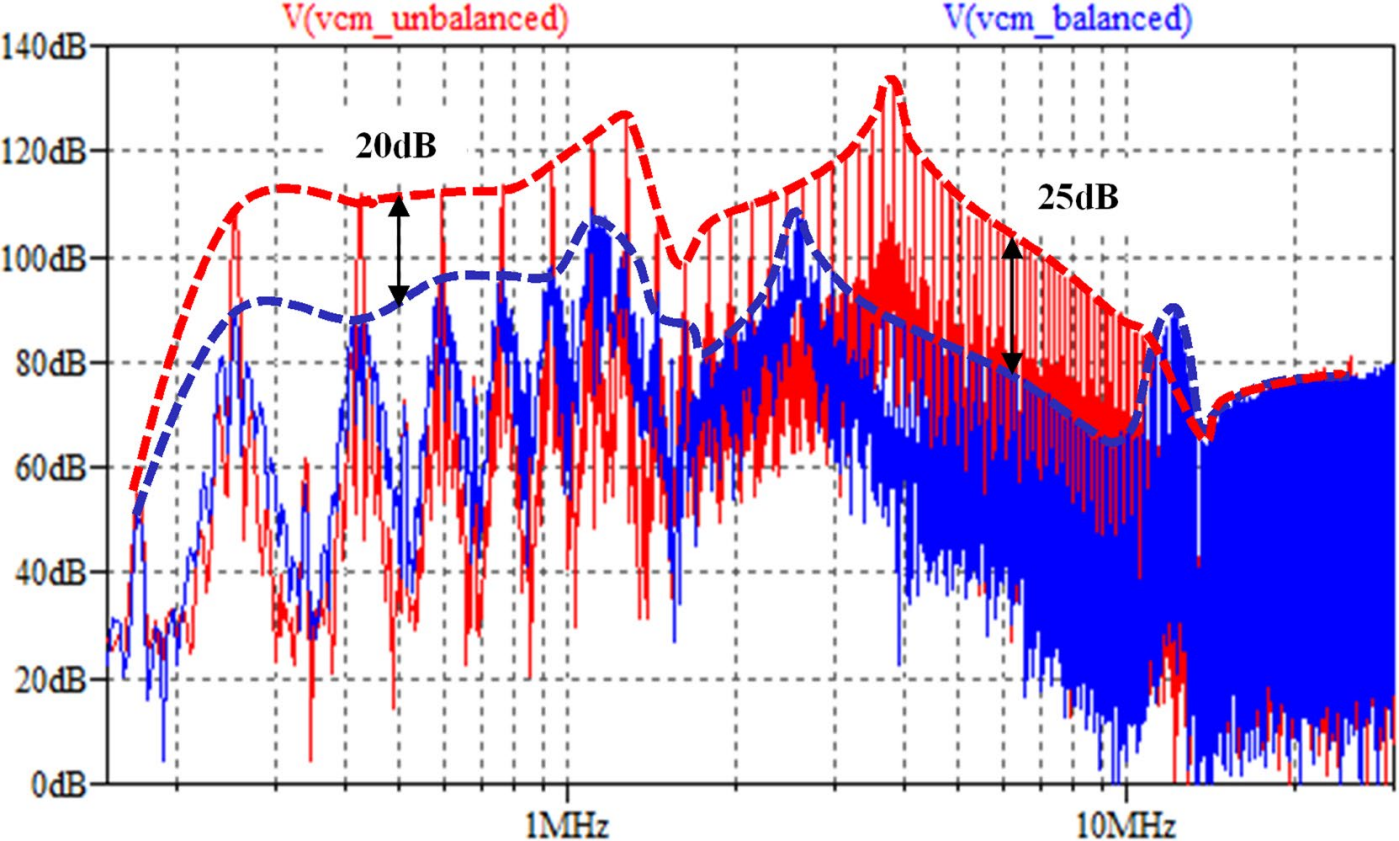
LTSpice-simulated CM noise results by two compensations.

The parasitic capacitances of IPT coils in Table 2 and two sets of compensation networks in Table 3 are applied to conduct the simulation. The CM noise simulation results are displayed in Fig. 16. It clearly shows that a balanced impedance design can deliver over 20 dB CM noise attenuation below the 10 MHz frequency spectrum.

## Experimental validation

In this section, the experimental setup and EMI test results of an 11 kW IPT prototype are provided, and two sets of compensation network hardware are employed to verify the performance of the proposed balance technique.

### Experimental prototype

**Fig. 17:**
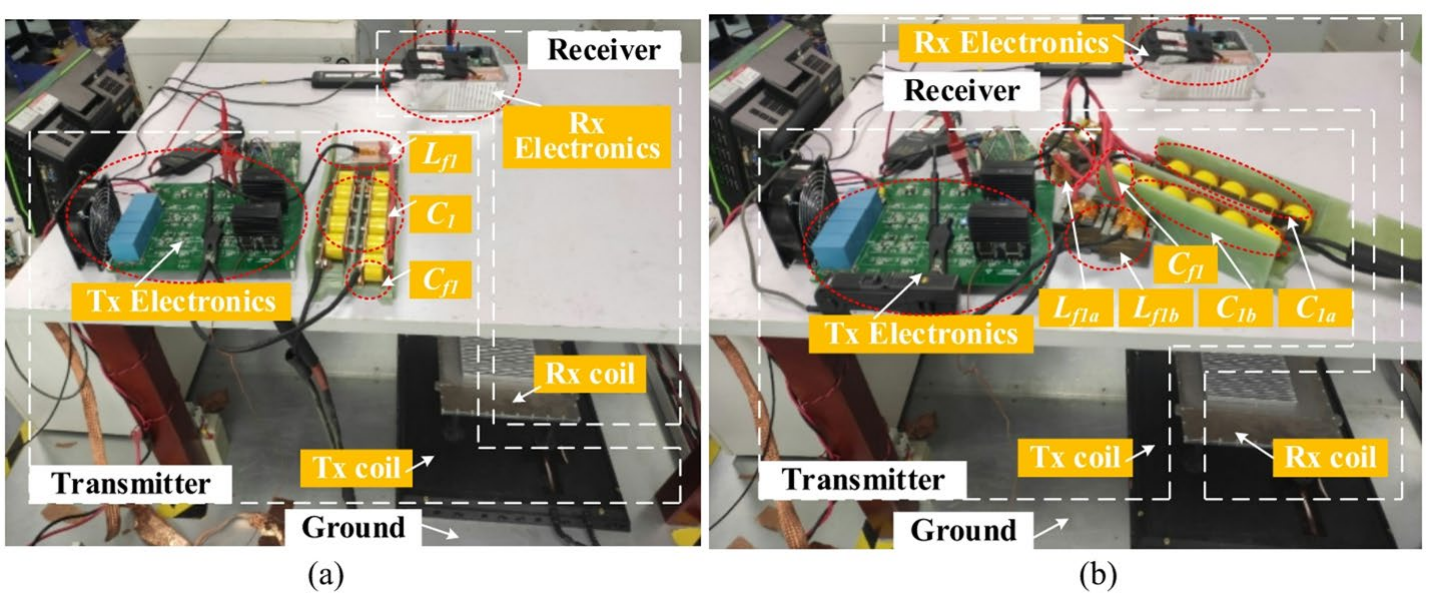
Experimental prototype of an 11 kW IPT system. (**a**) with unbalanced impedance network; (**b**) with balanced impedance network.

Conductive EMI tests are performed on an 11 kW IPT prototype, which consists of four parts: the transmitter coil, the receiver coil, transmitter electronics, and receiver electronics, demonstrated in Fig. 17. Table 4 lists the circuit parameters and devices in the experimental prototype.

**Table 4:** Circuit parameter and devices in the IPT prototype.

Parameter or device	Symbol	Value or model
Input voltage	*V*_*BUS*_	350–700 V
Output voltage	*V*_*BAT*_	280–420 V
Switching frequency	*f*_*sw*_	85 kHz
Coil distance	*Z*_*gap*_	140 ~ 210 mm
Output power	*P*_*out*_	10 kW
Inverter switches	*S*_*1*_*, S*_*2*_*, S*_*3*_*, S*_*4*_	C2M0025120D
Rectifier diodes	*D*_*1*_*, D*_*2*_*, D*_*3*_*, D*_*4*_	RURG5060_F085

The transmitter electronics is composed of a control board, an inverter board, and a set of resonant components. Two types of resonant components are employed in experimental tests: one is the conventional LCC compensation network with unbalanced CM impedance; the other one is an improved symmetrical LCC network with balanced CM impedance, as shown in Fig. 17a and b, respectively.

**Fig. 18:**
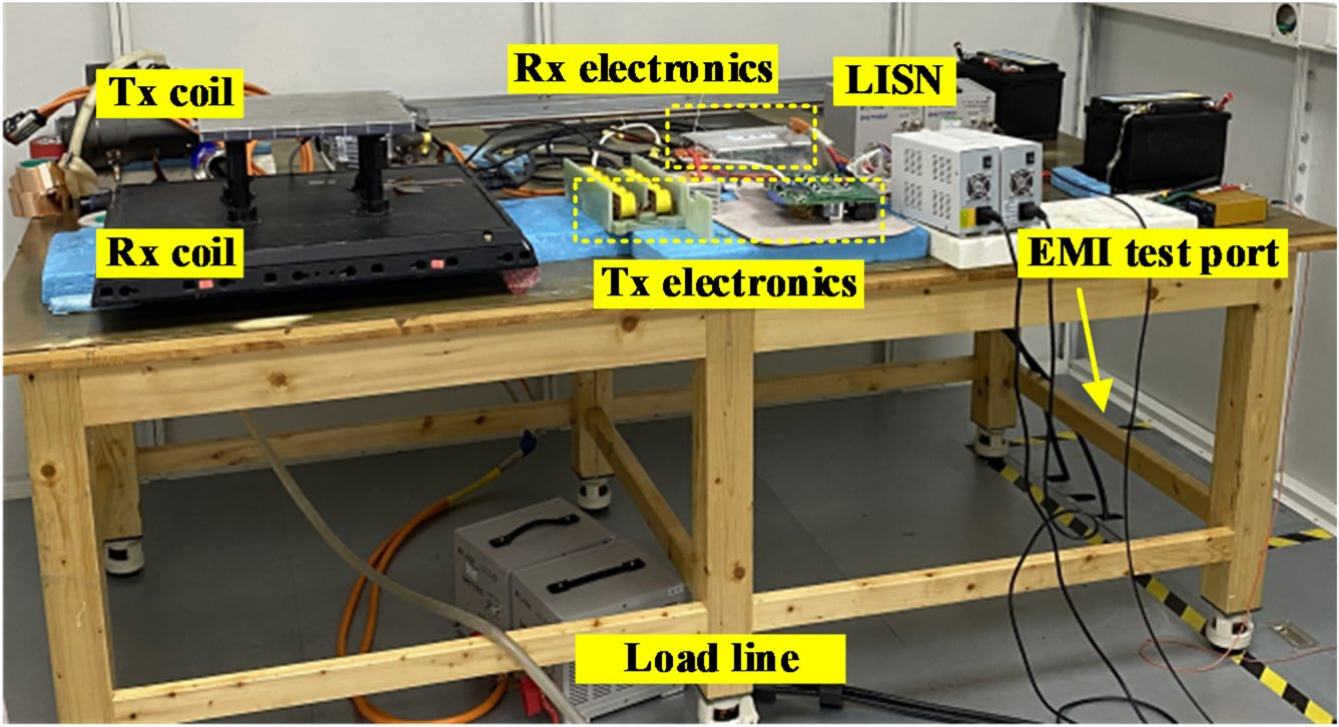
Conductive EMI test platform.

To validate the proposed CM noise model and balance technique, the effectiveness of CM noise suppression is evaluated through a standard EMI test. Figure 18 demonstrates the structure and apparatus of the conducted EMI test platform. The setup follows the CISPR 22 standard for conductive EMI testing, covering the frequency range from 0.15 MHz to 30 MHz. The transmitter and receiver coils with their associated electronic components are mounted on a wooden table positioned 80 cm above the ground. Additionally, the electronics and coils are located at least 80 cm from the LISN equipment.

Both the DC power source and the LISN equipment comply with the standard requirements. The LISN used is CYBERTEK/EM5040B with the CM & DM separator built-in. The EMI receiver is Rohde & Schwarz, R&S ESL3/ESL6.

### EMI testing results

The IPT device is powered by a programmable DC source during experimental testing and the charging current sinks to a programmable DC load. The IPT system is able to transfer 10 kW power when the receiver coil is well-aligned with the transmitter coil. The detailed parameters of the coils are listed in Table 1. The design of the LCC compensation network is listed in Table 2, which is utilized to achieve soft switching. The device parameters are listed in Table 4.

**Fig. 19:**
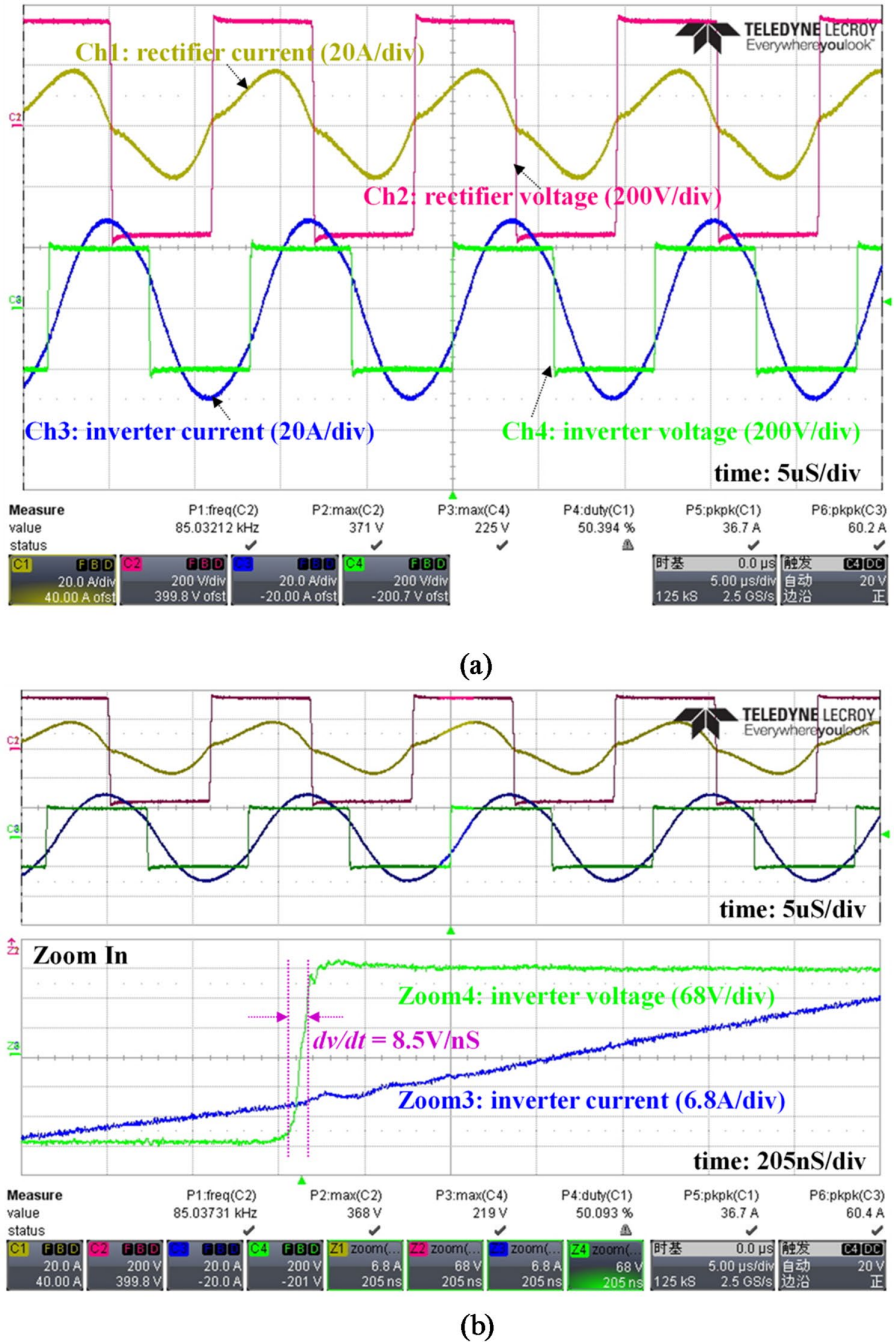
Experimental waveforms. (**a**) current & voltage of inverter and rectifier; (**b**) zoom in review of switching transient.

Figure 19a shows the typical waveforms of inverter output current and voltage during power transfer. It clearly shows that a square-wave voltage excitation is provided and a sinusoidal output current is generated. Based on the current waveform, the zero-voltage-switching (ZVS) turn-on is realized for MOSFETs in the inverter. Figure 19b is the zoom-in review of inverter waveforms at the switching moment, which shows that the dv/dt of inverter voltage is up to 8.5 V/nS.

In the conductive EMI tests, the prototype was evaluated under three cases: ungrounded, grounded without an EMI filter, and grounded with an EMI filter. These cases influence the EMI performance under the same test setup described in Section "EMI testing results". The grounding configuration directly affects the stray capacitance, which plays a critical role in CM noise behavior. The EMI filter, shown in the schematic of Fig. 9, consists of CM chokes and Y-capacitors and is placed between the LISN and the inverter. The balance technique, realized through the symmetrical compensation circuit, is evaluated across these three test cases.

**Fig. 20:**
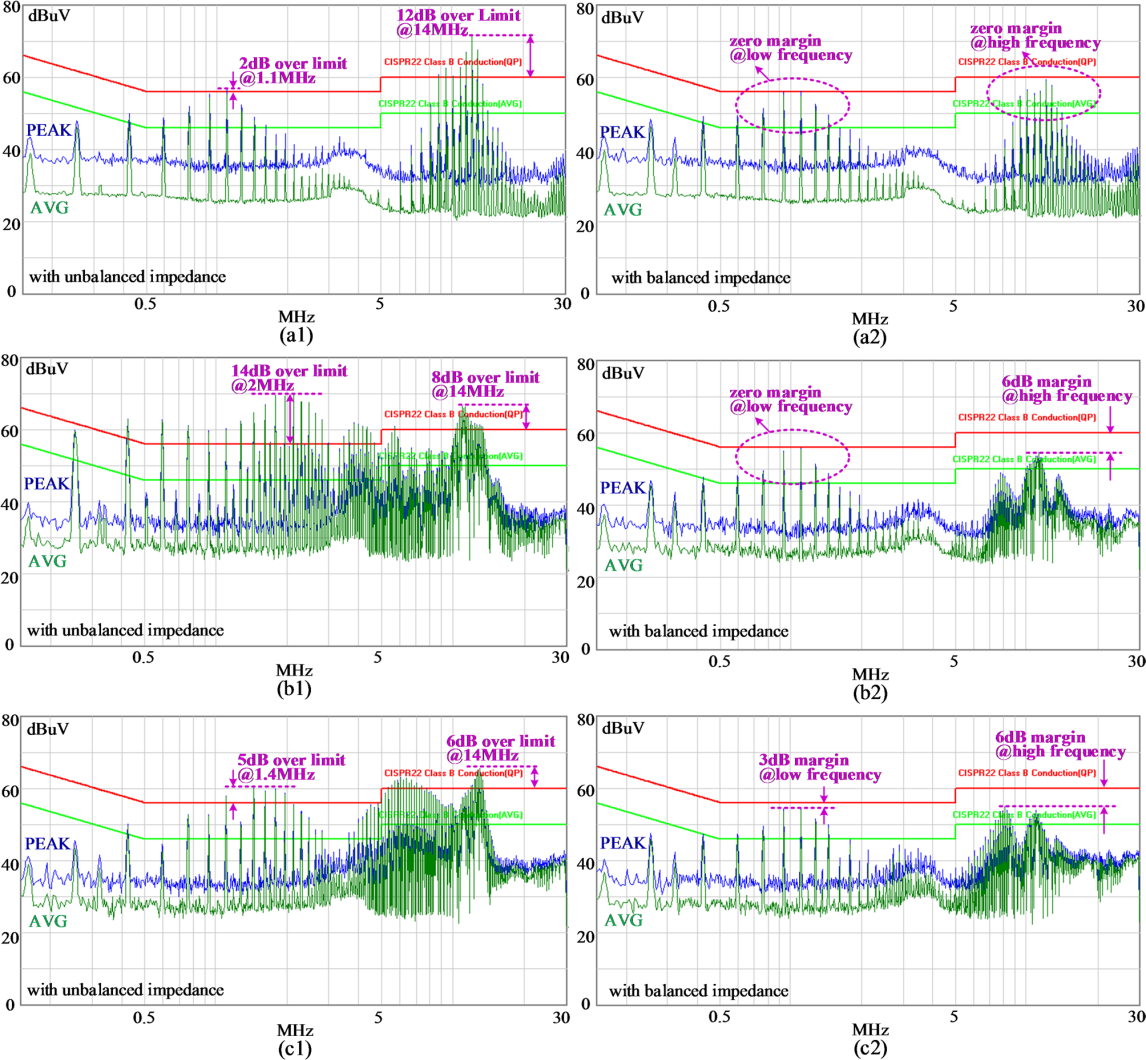
Conductive CM EMI test results. (**a1**) “ungrounded case”*,* with unbalanced impedance*.* (**b1**) “grounded case”, without an EMI filter*,* with unbalanced impedance*.* (**c1**) “grounded case”, with an EMI filter*,* with unbalanced impedance. (**a2**) “ungrounded case”*,* with balanced impedance*.* (**b2**) “grounded case”, without an EMI filter*,* with balanced impedance*.* (**c2**) “grounded case”, with an EMI filter*,* with balanced impedance.

Figure 20(a1), (b1) and (c1) present the conductive EMI test results of the original setup with unbalanced CM impedance. Each subfigure corresponds to different cases. In Fig. 20(a1), the metal shielding layer of the Tx coil is not grounded. In Fig. 20(b1), the metal shielding layer of the Tx coil is grounded without an EMI filter. In Fig. 20(c1), the EMI filter is inserted in the IPT system. As observed, CM noise increases significantly after grounding. A comparison between Fig. 20(b1) and Fig. 20(c1) indicates that the CM noise is effectively reduced by the EMI filter, though elevated levels persist in certain frequency ranges: 5 dB over the limit of CISPR 22 Class B in the middle frequency spectrum, and 6 dB over the limit in the high-frequency spectrum.

Figure 20(a2), (b2) and (c2) show the conductive EMI test results after applying the balance technique based on a symmetrical compensation circuit topology. The same three cases were tested. Compared to Fig. 20(a1), (b1), and (c1), the EMI performance in Fig. 20(a2), (b2), and (c2) is noticeably improved after applying the balance technique. In Fig. 20(b2), since no CM noise suppression components were used in the original setup (Fig. 20(b1)), the balanced impedance significantly enhances CM noise suppression, providing approximately 14 dB of noise reduction in both the low- and high-frequency ranges. This results in a larger margin. In Fig. 20(c1), the EMI filter contributes to CM noise reduction compared to Fig. 20(b1), and the additional application of the balance technique in Fig. 20(c2) further improves the suppression effect, providing approximately 8 dB of noise reduction in the low-frequency range and around 12 dB in the high-frequency range. By contrast, the improvement between Fig. 20(b2) and Fig. 20(c2) is relatively small, indicating that the balanced structure is more effective for CM noise suppression than a conventional EMI filter. These comparisons highlight that balancing the asymmetric CM impedance in the transmitter coil effectively mitigates CM noise throughout the system, which is the primary objective of this manuscript.

Table 5 compares the EMI suppression method proposed in this paper with those from referenced studies. In^4^, an improved filter was designed using a genetic algorithm, based on real voltage and current data from the inverter system. This filter reduces noise by over 20 dB across the full frequency range compared to a conventional filter. It uses six passive components and the design is complex, but it operates without advanced control. Xie et al.^8^ cancels CM voltage by injecting a transformer between both the input and output CM inductors in the inverter. Compared to using only CM inductors, the added transformer improves CM current suppression by up to 40 dB at low frequencies and by 5-10 dB at high frequencies. The design uses two CM inductors and a pair of cancellation windings, forming a simple structure that requires no control. Han et al.^11^ suppresses EMI by coordinating the control of multiple half-bridge converters in a series-input parallel-output system. Compared to conventional control strategy, it achieves up to 50 dB noise reduction at low frequencies, though improvements at high frequencies are minimal. This approach requires no extra filtering components and is well-suited for systems with complex inverter structures.

**Table 5:** Comparisons of EMI suppression methods.

Papers	Methods		Conductive EMI suppression
	Added components	Design complexity	
^4^	A Filter with two inductors and four capacitors	Genetic algorithms	20dBuV@Overall
^8^	Two CM inductors and a cancellation CM transformer	Negligible	Over 40dBuA@low 5 ~ 10dBuA@high
^11^	Negligible	Detected currents and voltages for coordinated control in multiple half-bridge cells	50dBuV@low 0dBuV@high
This paper	Negligible	Negligible	8dBuV@low 12dBuV@high

In this work, the compensation capacitors in high-power IPT systems are typically implemented using multiple units to meet voltage withstand requirements. Hence, separating these capacitors does not require additional components. Furthermore, this method places no constraints on the control strategy. Compared to unbalanced bridge configurations, it achieves an extra 8 dB suppression at low frequencies and up to 12 dB at high frequencies.

## Conclusion

In this paper, a high-power IPT system is studied considering the CM impedance. A distributed model of the coil is presented to reveal the uneven stray capacitances with respect to ground, thereby a simplified circuit of the asymmetric CM impedance at two coil terminals is obtained. Based on this, the CM noise model of a high-power IPT system is derived. By adjusting the existing compensation network of the coil, the asymmetric CM impedance at its two ports can be balanced. The balance technique with a symmetric compensation network is proposed to mitigate conductive CM noise. The design method of the balanced impedance is provided and the LCC compensation network is utilized as an example. According to the EMI test results in an 11 kW IPT prototype, the balance technique could significantly suppress the CM noise and keep it below the limit of the CISPR 22 standard, which is of great significance for high-power-based wireless power and information transmission.

This paper provides an analytical model for the CM noise impedance and a design method of the balance technique in the LCC compensation network. The same methodology can be extended to other compensation topologies. Moreover, the CM noise model proposed in this paper disregards the influence of the secondary coil due to the large coil gap. In other coupling structures, the CM noise circuit model should be improved based on the actual characteristics of the parasitic parameters between the coils.

## Data Availability

All data generated or analyzed during this study are included in this article.
